# Financial development and natural resources for the top five gas exporters

**DOI:** 10.1016/j.heliyon.2023.e20273

**Published:** 2023-09-19

**Authors:** Mihaela Simionescu, Beata Gavurova

**Affiliations:** a Faculty of Business and Administration, University of Bucharest, Bucharest, Romania; b Institute for Economic Forecasting, Romanian Academy, Bucharest, Romania; c Faculty of Mining, Ecology, Process Control and Geotechnologies, Technical University of Košice, Košice, Slovakia

**Keywords:** Resource curse, Financial development, Natural resources, Panel data

## Abstract

Given the recent preoccupations of scientific environment for “resource curse” in the context of financial development, the aim of this paper is to explore more the connection between financial development and natural resources abundance/dependence in the major five gas exporters in the period 1996–2021. The panel data models based on DOLS/FMOLS approach and mean group (MG) estimator suggest that the impact of resource abundance on financial development depends on the type of indicators used to assess the financial progress, as previous studies suggested. More coal per capita and the interaction between contract intensity and total natural resources, respectively oil per capita contribute to the development of financial markets, while economic growth supports the development of financial institutions. Even if natural gas per capita is causally related to financial development, it does not exert a significant impact on it in these countries. The new international context might be an opportunity for US, Canada, China and Saudi Arabia to achieve this target.

## Graphical abstract

**Figure:**
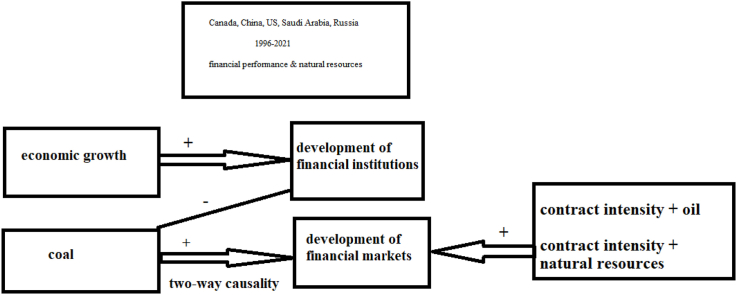

## 1 Introduction

The corporations can impact growth due to its influence on financial, human and natural resources. A well-developed financial system is necessary for corporations that extract natural resources and ensure human resources to explore and process natural resources [1]. On the other hand, revenues and rents determined by natural resources are directed to economy and support economic growth. The connection between resource, finance and growth nexus has been widely addressed in the previous studies considering natural resource and financial resource curses, but the results are still inconclusive.

The recent framework in policy and development is based on the central concept of “resource curse” that is related to lagging financial performance and abundant natural resources. The first definition is provided by Auty and Warhurst and it reflects the negative connection between natural resource abundance and growth [2]. However, next advances in the literature associate the concept with “Dutch disease”. The latter shows the rent-seeking behavior of influential powerful individuals that use revenues of natural resources for their advantage and break the infrastructure development. Resource endowment is also discussed from another perspective. Natural resources are determinant, but the development of financial systems is conditioned by laws of each country. Natural resources refer to forests, oil, gas, and minerals that are available in a country [3].

The traditional approach states that natural resources have a positive contribution to one country economic development. However, the empirical evidence shows that many resource-abundant economies performed less efficient with a slower economic growth rate compared to resource-poor economies [4]. This situation is known as the “natural resource curse”. Next studies focused on explanations of these unexpected results. Gylfason identified four economic channels that could explain the natural resource curse.

1. The boom in natural resources determines currency appreciation because of exchange rate volatility which is a sign for Dutch disease that affects exports from other sectors;
2. More rent-seeking activities expressed as corruption intensification, import protection among other privileges, inefficient resource allocation;
3. Inefficient management at national level caused by managerial overconfidence on resource abundance;
4. Nations with resource abundance might neglect investments in the education sector [5].

Besides these channels, further studies showed that financial channels are essential in the path of revenues determined by export of natural resources with direct impact on economic growth [6]. Moreover, the four channels described above are also connected to financial development since might break or slower the financial development [7]. Financial development is related to that financial system with minim costs in the allocation of resources, savings and trade promotion for investment projects and diversified risk [8]. The common indicators used to measure financial development do not take into account the comprehensive dimensions associated to financial information. Therefore, this study proxies financial development through specific indicators like Financial development index, Financial institution index and Financial markets index [9].

The financial resource curse hypothesis was checked by Beck who showed that resource-abundant countries present lower financial development [10]. The author showed that the connection between resource-abundance and financial development might be analyzed from demand and supply point of view. On demand-side, more demand for financial services grows consumer credit because of Dutch disease. On supply-side, deterioration of investment and skills in financial sector is caused by resource abundance in a country.

The examination of the natural resources-financial development nexus serves three main purposes. First, it presents a unique empirical approach to understanding this nexus. Natural resources have direct effect on financial development and stimulate economy by providing financial services for the development of the capital projects. The connection between growth, natural resources, and finance can have both direct and indirect impact, depending on the specific characteristics of the resources and the economy [8]. Second, exploring the connection between natural resources and financial development offers valuable insights into different types of areas, including developing, emerging and developed economies with different stages of financial development. This analysis enhances our understanding of how a country's natural resource availability influences the development of its financial sector. Lastly, natural resources-financial development nexus is essential for policymakers and governments. It provides empirical evidence to support the formulation of good economic policies that utilize natural resources as a tool to promote economic growth and enhance financial development within a region [6].

Natural resources have the potential to enhance financial development through various channels. First, the extraction of natural resources often leads to a shift of factors of production away from the manufacturing sector. This can limit a country's exports, and export diversity is crucial for promoting financial development. Conversely, an abundance of natural resources can hinder financial development by reducing a country's exports [10].

Second, the extraction of natural resources can contribute to rent-seeking behavior. This tends to decrease the number of industrialists, which negatively impacts financial development. Therefore, having an excessive amount of natural resources can be detrimental to a nation's financial development if rent-seeking is not addressed [5].

Third, abundance of natural resources not only stimulates growth but also improves financial development. This is a significant factor to consider when examining financial development, as economic growth is greatly influenced by the availability of natural resources. Consequently, natural resources can contribute to financial development through their impact on economic growth and production [7].

Fourth, the combination of export diversity, natural resources, and growth can further enhance financial development. A well-developed financial sector aids nations in maintaining price stability, which in turn promotes stable inflation levels. Therefore, the interplay between natural resources, export diversity, economic growth, and financial development is crucial [10].

Lastly, it is important to note that financial development is also influenced by factors such as growth, export diversity, price growth, and natural resources. Any changes in these factors can negatively impact financial development, leading to reduced financial resources and services. Consequently, financial development can decline as a result [5].

In summary, natural resources can have both direct and indirect impact on financial development through different channels, including their impact on exports, rent-seeking behavior, economic growth, export diversity, and inflation levels. Understanding these complex relationships is essential for promoting sustainable financial development.

The relationship between natural resource and financial development has only recently attracted the interest of researchers and the results are still inconclusive because of the specific characteristics of social, political, legislative and cultural environment. Asif et al. and Yuxiang and Chen showed that natural resources stimulate financial development that is the main driver of long-run economic growth [11,12]. Other papers indicated that growth enhances financial development in the US [6] and China [13]. For G7 states, Wei et al.showed a positive relationship between natural resources rent and the financial development index in the period 1990–2021 [14]. For more developed countries, Dogan et al.supported the hypothesis that natural resources determined financial development and economic growth using quantile regression in the period 2001–2021 [15]. Many other studies concluded that resource abundance in some countries is associated with low levels of financial development in 85 countries [16], 101 countries in period 1990–2014 [17], 87 Emerging and Developing Economies [7], Malaysia [18] in the case of oil dependence, Pakistan during 1970–2018 in the case of natural resource rents-broad money supply nexus [19]. However, there are cases when no connection is identified. For example, for 38 African countries, Dwumfour and Ntow-Gyamfishowed that the relationship between financial development and natural resources is ambiguous. On the other hand, a short-run and long-run perspective might be more informative [20]. For example, for G7 states, Li et al. indicated a negative impact of natural resources on financial development in the short-run and a positive one in the long-run [21]. The financial resource curse hypothesis was validated by Tang et al. for ASEAN states in the interval 1984–2018 and business regulations proved to reduce the negative effects of natural resources on financial development [22]. For G7 countries in the period 1990–2020, natural resources have a negative effect on financial development [23].

**Fig. 1:**
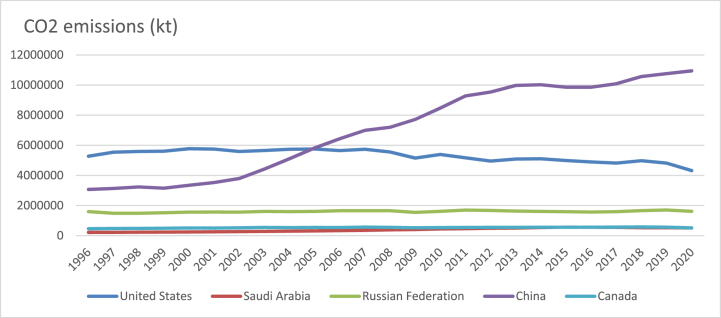
CO2 emissions (kt) in US, Canada, Russia, China and Saudi Arabia (1996–2020).

Source: own graph in Excel based on World Bank data

Aspects related to institutional quality, technological innovation, and human capital resources reflected by knowledge and skills of individuals are part of the natural endowment theory [7]. Previous studies are limited only to the analysis of one country like US, China etc. and the results of mixed: only 20% studies validated the positive connection between financial development and institutional quality, while 40% of them indicated a negative correlation. The remaining studies provided inconclusive results. Natural resources endowment is closely related to pollution and economic activities. In the sample of countries composed by China, US, Saudi Arabia, Russia and Canada, two observations could be drawn in the period 1996–2020: according to Fig. 1, in the interval 1996–2004, US was the country with the highest level of CO2 emissions, while since 2005, China is the leader in terms of pollution and the ascending tendency seem to keep.

Considering the scientific achievements in this field and the recent preoccupations of researchers for resources abundance and financial development, this paper brings some novel contributions. First, the paper assesses the impact of resource abundance and resource dependence on financial development using various proxies for dependent variable: financial development index, financial institution index and financial markets index. Resource dependent measures are represented by total natural resource rents, coal rents, oil rents and natural gas rents. Second, unlike other studies from literature, the analysis does not focus on one country. It refers to the top five gas exporters in the period 1996–2021: USA, Russia, Canada, China and Saudi Arabia. In the current international context characterized by the Russian invasion in Ukraine, gas export is a critical issue and the impact of resource abundance on these countries financial development is important to anticipate possible scenarios in the new context. Third, compared to previous studies, this paper employs more types of models: basic models, models based on contract intensity and models based on interaction term between contract intensity and natural resource abundance. Under cointegration between variables in panel data, DOLS/FMOLS approach is used. Robustness analysis is conducted using panel autoregressive distributed lag model (panel ARDL) in the form of mean group estimator (MG).

The results indicated a positive impact of GDP per capita only on financial institution index. As expected, broad money has a positive influence on all financial development indicators. Coal per capita negatively affects financial institution index, but positively impacts financial markets index. Contract intensity and its interaction with oil per capita contributes to increase in financial development index, while only the interaction between contract intensity and oil per capita, and total natural resource per capita respectively positively impact financial markets index. Market capitalization reduces financial institution index, but increases financial development index. Even if natural gas per capita does not have a direct influence on financial development indicators, bidirectional causality was observed between natural gas per capita and each financial markets indicator. There is also a reciprocal causality between contract intensity and financial development index, and financial institution index respectively.

In conclusion, the impact of resource abundance on financial development depends on the type of indicators used to assess the financial progress as previous studies suggested [7]. Even if natural gas per capita is causally related to financial development, it does not exert a significant impact in these countries. The new international context might be an opportunity for US, Canada, China and Saudi Arabia to achieve this target.

## 2 Methodology and data

The methodology is based on panel data models, but preliminary tests are necessary before establishing the suitable type of model. The variables introduced in this analysis are presented in Table 1. The data series refer to the period 1996–2021 and to the top 5 gas exporters: USA, Russia, Canada, China and Saudi Arabia. GDP per capita is computed by dividing GDP to total population of each country. There is no source for the abundance measures, they were constructed using World Bank inputs. Resource abundance measures in terms of value are.

● TNRA - total natural resource per capita (using GDP constant 2015 US$ and total population)
● CA – coal per capita (using GDP constant 2015 US$ and total population)
● OA – oil per capita (using GDP constant 2015 US$ and total population)
● NGA – natural gas per capita (using GDP constant 2015 US$ and total population)

**Table 1:** Variables’ description.

Variable	Notation	Definition	Source of data
Financial development index	FD	relative ranking of countries on depth, access and efficiency of their financial institutions and financial markets. It is an aggregate of the financial institutions index and financial markets index	IMF https://data.imf.org/?sk=F8032E80-B36C-43B1-AC26-493C5B1CD33B&sId=1480712464593
Financial institution index	FI	an aggregate of: **financial institutions depth index** (complies data on bank credit to the private sector in percentage GDP, pension fund assets to GDP, mutual fund assets to GDP, and insurance premiums, life and non-life to GDP); **financial institutions access index** (complies data on bank branches per 100,000 adults and ATMs per 100,000 adults) and **financial institutions efficiency index** (complies data on banking sector net interest margin, lending-deposits spread, non-interest income to total income, overhead costs to total assets, return on assets and return on equity)	IMF https://data.imf.org/?sk=F8032E80-B36C-43B1-AC26-493C5B1CD33B&sId=1480712464593
Financial markets index	FM	an aggregate of: **financial markets depth index** (complies data on stock market capitalization to GDP, stocks traded to GDP, international debt securities of government to GDP and total debt securities of financial and non-financial corporations to GDP); **financial markets access index** (complies data on percentage of market capitalization outside of top 10 largest companies and total number of issuers of debt per 100,000 adults) and **financial markets efficiency index** (complies data on stock market turnover ratio).	IMF https://data.imf.org/?sk=F8032E80-B36C-43B1-AC26-493C5B1CD33B&sId=1480712464593
Contract intensity	CI	institutionalization of asynchronous contract flows in countries.	https://dataverse.harvard.edu/dataset.xhtml?persistentId=doi:10.7910/DVN/8RPC9E
Gross domestic product (constant 2015 US$)	GDP	sum of gross value added by all resident producers in the economy plus production tax minus subsidies not included in product value. Data is expressed in dollar figures.	World Bank national accounts data
Domestic credits to private sector as % of GDP	DC	financial development measure– financial resources provided to the private sector by financial corporations through loans, purchase of non-equity securities, trade credits and accounts receivable, establish a claim of repayment.	IMF, international financial statistics and data files and World Bank
Broad money as % of GDP	BM	financial development measure – sum of currency outside banks; demand deposits and foreign currency deposits of resident sectors; banks and travelers checks; and other securities.	IMF, international financial statistics and data files and World Bank
Market capitalization of listed domestic companies as % of GDP	MK	financial development measure – share price times number of shares outstanding for listed domestic companies, end year values.	World federation of exchange database (coming from World Bank).
Population total	POP	all residents disregarding legal status or citizenship, midyear estimates.	United Nations population division (coming from World Bank)
**RESOURCE DEPENDENT MEASURES**			
total natural resource rents as % of GDP	TNR %GDP	sum of oil, coal, natural gas rents, mineral rents and forest rents.	World Bank
coal rents as % of GDP	CR %GDP	difference between value of coal production at world prices and their total cost of production.	World Bank
oil rents as % of GDP	OR %GDP	difference between value of crude oil production at regional prices and total cost of production.	World Bank
natural gas rents as % of GDP	NGR %GDP	difference between value of natural gas production at regional prices and total cost of production.	World Bank

**Fig. 2:**
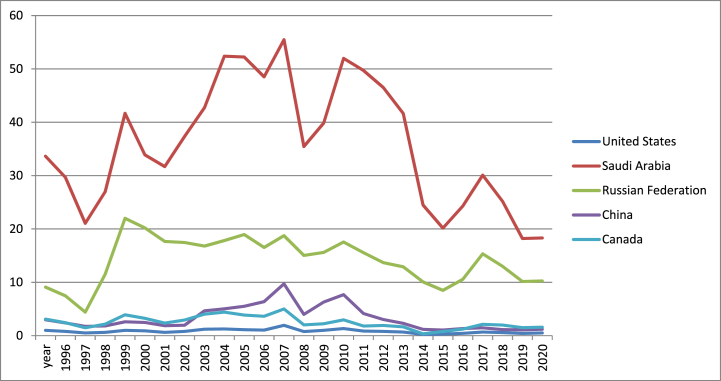
Total natural resource rents (% of GDP) in US, Canada, Russia, China and Saudi Arabia (1996–2021).

Source: own graph in Excel based on World Bank data

In the following figures, the evolution of few indicators is described for the five countries (United States, China, Canada, Russia and Saudi Arabia) in the period 1996–2021. The values for 2021 are imputed using extrapolation. According to Fig. 2, Saudi Arabia registered the maximum values for share of total natural resource rents in GDP, while the US recorded the minimum values. However, Saudi Arabia that is rich in natural resources registered lower economic growth compared to US. Canada is natural resource-intensive economy that has registered constant economic performance for a long period.

**Fig. 3:**
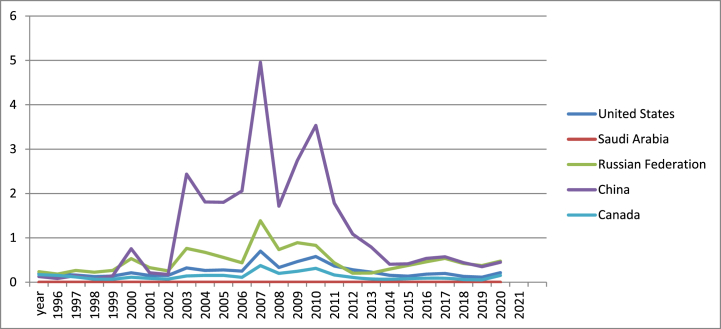
Coal rents (% of GDP) in US, Canada, Russia, China and Saudi Arabia (1996–2021).

Source: own graph in Excel based on World Bank data

According to Fig. 3, since 2004, China is the leader in terms of coal rents, while the absence of coal in Saudi Arabia makes this country the one with the least performance. Actually, China is the largest producer and consumer of coal in the world, this resource being used on large scale to produce electricity.

**Fig. 4:**
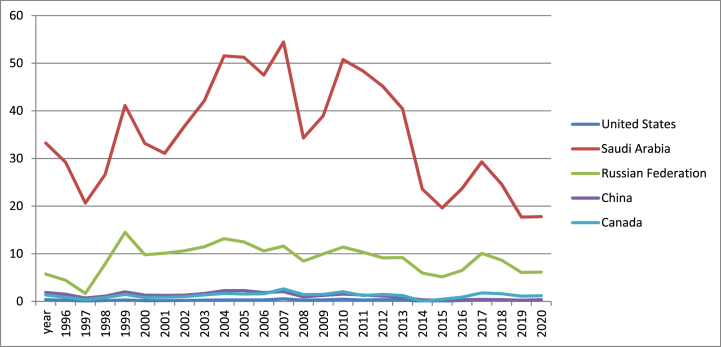
Oil rents (% of GDP) in US, Canada, Russia, China and Saudi Arabia (1996–2021).

Source: own graph in Excel based on World Bank data

Fig. 4 shows that Saudi Arabia presents the maximum values of oil rents due to the proven reserves of oil that place this country as the second in the world after Venezuela. On the other hand, the minim values for this indicator were recorded by the US.

**Fig. 5:**
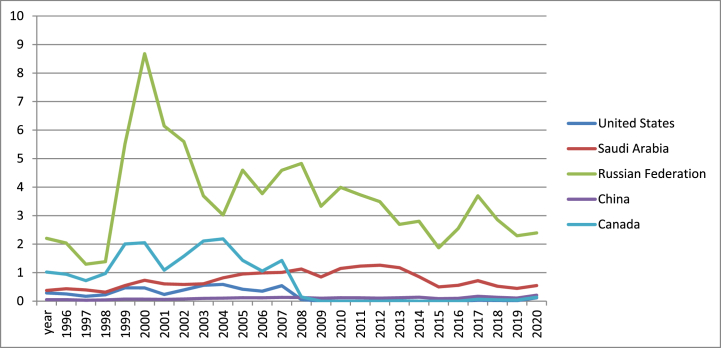
Natural gas rents (% of GDP) in US, Canada, Russia, China and Saudi Arabia (1996–2021).

Source: own graph in Excel based on World Bank data

Fig. 5 indicates that Russian Federation is leader in terms of natural gas rents, while the minimum values were registered by China (1996–2008) and US (2009–2021). Since 2013, Russia is the second largest producer of natural gas after Iran.

**Fig. 6:**
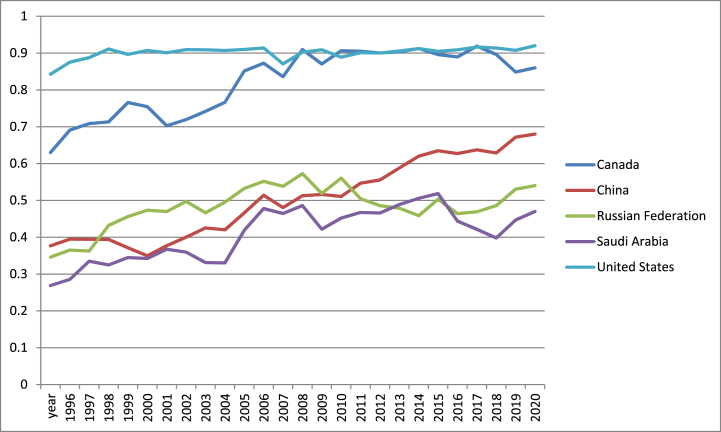
Financial development index in US, Canada, Russia, China and Saudi Arabia (1996–2021).

Source: own graph in Excel based on IMF data

The highest values for financial development index are observed in the US excepting few years, when Canada reached the maxim (2009, 2011–2013, 2015, 2018) as Fig. 6 shows. On the other hand, Saudi Arabia showed the least performance excepting the period 2014–2016 when Russia registered the minimum values for financial development index. Actually, Saudi Arabia does not have an effective financial sector to support the economic growth, while US developed many markets oversea that support the export.

**Fig. 7:**
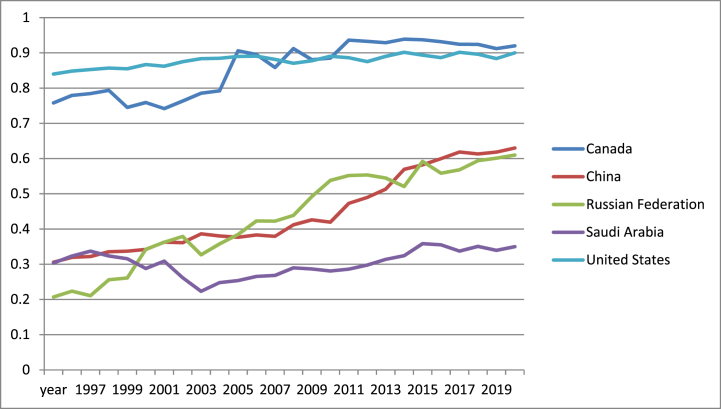
Financial institutions index in US, Canada, Russia, China and Saudi Arabia (1996–2021).

Source: own graph in Excel based on IMF data

According to Fig. 7, the maximum values of financial institutions index were registered by US in the period 1996–2005 and in the years 2008 and 2011. In the rest of the period, Canada had the highest values for this indicator due to strong policy directives. Actually, Canada was among the countries that had institutions which manage very well the Covid-19 crisis. On the other hand, in the period 1996–2000, Russian recorded the minimum values for financial institutions index and in the rest of the period Saudi Arabia proved the weakest performance.

**Fig. 8:**
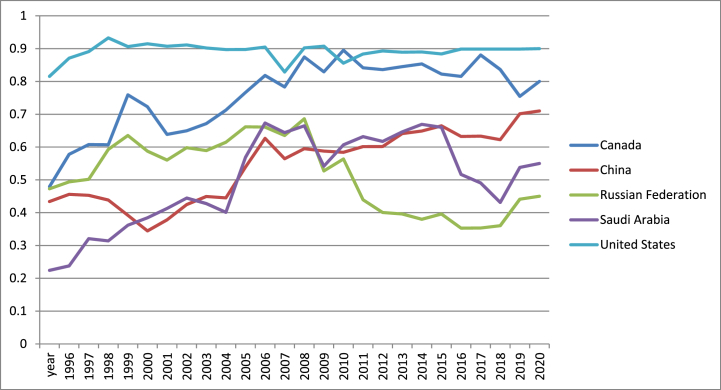
Financial markets index in US, Canada, Russia, China and Saudi Arabia (1996–2021).

Source: own graph in Excel based on IMF data

According to Fig. 8, US registered the highest values of financial market index in the entire period, excepting 2011. These values are followed by investors that take decisions of investing in a market after analyzing the performance of the economy reflected by this index. Starting with 2010, Russian Federation recorded the minimum values of this index which makes Russia less attractive for investors. Moreover, the actual military conflict between Ukraine and Russia will make the latter even less attractive for foreign investors.

All the data series are considered in natural logarithm to have an interpretation in terms of elasticities. The research starts from a baseline model that is then extended for robustness check. The dependent variables are represented by: financial development index, financial institutions index, and financial market index.

FD_*i,t*_ = *α growth*_*i,t*- 1_ + *ANaturalResourseAbundance*_*i*_*,*_*t*- 1_ + *υZ*_*i,t*- 1_ + *δ*_*i*_ + *ω*_*t*_ + *μ*_*i,t*_

FI_*i,t*_ = *α growth*_*i,t*- 1_ + *ANaturalResourseAbundance*_*i*_*,*_*t*- 1_ + *υZ*_*i,t*- 1_ + *δ*_*i*_ + *ω*_*t*_ + *μ*_*i,t*_

FM_*i,t*_ = *α growth*_*i,t*- 1_ + *ANaturalResourseAbundance*_*i*_*,*_*t*- 1_ + *υZ*_*i,t*- 1_ + *δ*_*i*_ + *ω*_*t*_ + *μ*_*i,t*_

FD_*i,t*_ = *α growth*_*i,t*- 1_ + *βNaturalResourceDependence*_*i,t*- 1_ + *ϑ Z*_*i,t*- 1_ + *γ*_*i*_ + *∂*_*t*_ + *ε*_*it*_

FI_*i*_*,t = α growthi,t- 1 + βNaturalResourceDependencei,t- 1 + ϑ Zi,t- 1 + γi + ∂t + εit*

*FMi,t = α growth*_*i,t*- 1_ + *βNaturalResourceDependence*_*i,t*- 1_ + *ϑ Z*_*i,t*- 1_ + *γ*_*i*_ + *∂*_*t*_ + *ε*_*it*_

● *NaturalResourseAbundance*: the log of natural resource value per capita
● *NaturalResourceDependence*: natural resources value as % of GDP
● *Z:* a vector of control variables:
● *δ, γ:* country-fixed effects
● *ω, ∂:* time-fixed effects that capture common shocks to FD for all countries.

For robustness, contract intensity (CI) is included in the models and the interactions between contract intensity and natural resource abundance. The new models are presented below.

### 2.1 Models based on contract intensity

FD_*i,t*_ = *α growth*_*i,t*- 1_ + μCI + *A(NRA)*_*i,t*-1_ + *υZ*_*i,t*- 1_ + *δ*_*i*_ + *ω*_*t*_ + *μ*_*i,t*_ FI_*i,t*_ = *α α growth*_*i,t*- 1_ + μCI + *A(NRA)*_*i,t*-1_ + *υZ*_*i,t*- 1_ + *δ*_*i*_ + *ω*_*t*_ + *μ*_*i,t*_ FM_*i,t*_ = *α growth*_*i,t*- 1_ + μCI + *A(NRA)*_*i,t*-1_ + *υZ*_*i,t*- 1_ + *δ*_*i*_ + *ω*_*t*_ + *μ*_*i,t*_

FD_*t*_ = *α growth*_*i,t*-1_ + δCI+ *β(NRD)*_*i,t*- 1_ + *ϑ Z*_*i,t*- 1_ + *γ*_*i*_ + *∂*_*t*_ + *ε*_*it*_

FI_*t*_ = *α growth*_*i,t*-1_ + δCI+ *β(NRD)*_*i,t*- 1_ + *ϑ Z*_*i,t*- 1_ + *γ*_*i*_ + *∂*_*t*_ + *ε*_*it*_

FM_*t*_ = *α growth*_*i,t*-1_ + δCI+ *β(NRD)*_*i,t*- 1_ + *ϑ Z*_*i,t*- 1_ + *γ*_*i*_ + *∂*_*t*_ + *ε*_*it*_

### 2.2 Models based on interaction term

FD_*i,t*_ = *α growth*_*i,t*-__1_ + μCI +CI*NRA + *A(NRA)*_*i*_*,*_*t*- 1_ + *υZ*_*i,t*- 1_ + *δ*_*i*_ + *ω*_*t*_ + *μ*_*i,t*_

FI_*i,t*_ = *α growth*_*i,t*- 1_ + μCI +CI*NRA + *A(NRA)*_*i*_*,*_*t*- 1_ + *υZ*_*i,t*- 1_ + *δ*_*i*_ + *ω*_*t*_ + *μ*_*i,t*_

FM_*i,t*_ = *α growth*_*i,t*- 1_ + μCI +CI*NRA + *A(NRA)*_*i*_*,*_*t*- 1_ + *υZ*_*i,t*- 1_ + *δ*_*i*_ + *ω*_*t*_ + *μ*_*i,t*_

FD_*i,t*_ = *α growth*_*i,t*- 1_ + δCI +CI*NRD + *β(NRD)e*_*i,t*- 1_ + *ϑ Z*_*i,t*- 1_ + *γ*_*i*_ + *∂*_*t*_ + *ε*_*it*_

FI_*i,t*_ = *α growth*_*i,t*- 1_ + δCI +CI*NRD + *β(NRD)e*_*i,t*- 1_ + *ϑ Z*_*i,t*- 1_ + *γ*_*i*_ + *∂*_*t*_ + *ε*_*it*_

FM_*i,t*_ = *α growth*_*i,t*- 1_ + δCI +CI*NRD + *β(NRD)e*_*i,t*- 1_ + *ϑ Z*_*i,t*- 1_ + *γ*_*i*_ + *∂*_*t*_ + *ε*_*it*_

Preliminary tests should be applied before the selection of the most suitable type of model. A concise description of these tests is presented in Table 2. If cointegration is checked, DOLS/FMOLS approach is used. Otherwise, panel autoregressive distributed lag model (panel ARDL) is considered with mean group estimator (MG) as a particular case.

**Table 2:** The description of the preliminary tests before the construction of panel data models.

Test for:	Specific test	Null hypothesis of the test	Formulae	Advantages
cross-sectional dependence	**Pesaran's** (2004) CD test	cross-sectional independence	$CD=\sqrt{\frac{2T}{N\left(N-1\right)}}\sum_{i=1}^{N-1}\sum_{j=i+1}^{N}\frac{\left(T-k\right){\hat{\rho }}_{ij}^{2}-E\left[\left(T-k\right){\hat{\rho }}_{ij}^{2}\right]}{var\left[\left(T-k\right){\hat{\sigma }}_{ij}^{2}\right]}$ $Y_{it}=\alpha_{i}+\beta_{i}X_{it}+u_{it}$ i-country index, t- time index $X_{it}$- k x 1 vector of explanatory variables T-period length, N-number of cross-sections, $\hat{\rho_{ij}}$- estimated coefficient of pair-wise correlation using OLS	No distortions in the CD statistic because of small values for T and N
Slope heterogeneity	Pesaran and Yamagata (2008) test	slope homogeneity	$\tilde{S}=\sum_{i=1}^{N}{\left({\hat{\beta }}_{i}-{\tilde{\beta }}_{WFE}\right)}^{\prime }\frac{X_{i}^{\prime }I_{t}X_{i}}{{\tilde{\sigma }}_{i}^{2}}\left({\hat{\beta }}_{i}-{\tilde{\beta }}_{WFE}\right)$ $I_{t}$- identity matrix ${\tilde{\sigma }}_{i}^{2}$- variance estimator ${\hat{\beta }}_{i}$- slope OLS estimator for each cross-section *i* ${\tilde{\beta }}_{WFE}$- weighted fixed effect pooled estimator $\hat{\Delta }=\sqrt{N}∙\frac{N^{-1}\tilde{S}-k}{\sqrt{2k}}$ ${\bar{\Delta }}_{adj}=\sqrt{N}∙\frac{N^{-1}\tilde{S}-E\left({\bar{z}}_{it}\right)}{\sqrt{var\left({\bar{z}}_{it}\right)}}$ $E\left({\bar{z}}_{it}\right)=k$, $var\left({\bar{z}}_{it}\right)=\frac{2k\left(T-k-1\right)}{T+1}$ $\hat{\Delta }$- standardized variance ${\bar{\Delta }}_{adj}$ - biased-adjusted variance	Control of interactions between cross-sections to avoid unreliable estimation
Unit root	Covariate Augmented Dickey-Fuller (CADF) unit root test	Presence of unit root	$\Delta Y_{i,t}=\alpha_{i}+\beta_{i}Y_{i,t-1}+\gamma_{i}{\bar{Y}}_{t-1}+\delta_{i}\Delta {\bar{Y}}_{i,t}+e_{it}$ ${\bar{Y}}_{t-1}=\frac{1}{N}\sum_{i=1}^{N}Y_{i,t-1}$ $\Delta {\bar{Y}}_{i,t}=\frac{1}{N}\sum_{i=1}^{N}\Delta Y_{i,t}$	robust to cross-sectional dependence
cointegration	Westerlund test	No cointegration	$\Delta Y_{i,t}=\delta_{i}^{\prime }d_{t}+\rho_{i}\left(Y_{i,t-1}-\beta_{i}^{\prime }X_{i,t-1}\right)+\sum_{j=1}^{K}\varphi_{ij}Y_{i,t-j}+\sum_{j=0}^{K}\varphi_{ij}X_{i,t-j}+u_{i,t}$ $\rho_{i}$- speed of adjustment to equilibrium Group mean statistics • cointegration in at least one cross-sectional unit $G_{t}=\frac{1}{N}\sum_{i=1}^{N}\frac{\rho_{i}}{se\left(\hat{\rho_{i}}\right)}$ $G_{a}=\frac{1}{N}\sum_{i=1}^{N}\frac{T\rho_{i}}{\rho_{i}^{\prime }\left(1\right)}$ • cointegration in the entire panel $P_{t}=\frac{\hat{\rho_{i}}}{se\left(\hat{\rho_{i}}\right)}$ $P_{a}=T\hat{\rho }$	robust to cross-sectional dependence

The Fully Modified Least Square (FMOLS) was introduced by Phillips and Hansen to manage the estimation optimal of a co-integrating regression model [24]. This technique has the advantage of dealing with heterogeneous cointegration. This paper uses the heterogeneous FMOLS estimator of Pedroni that corrects autocorrelation and endogeneity bias [25]. Let us start from a cointegrated system (equation (1) and (2)) in the case of a panel with N cross-sections (i = 1,2,..,N):

(1) $$y_{it}=\alpha_{i}+\beta x_{it}+\mu_{it}$$

(2) $$x_{it}=x_{it-1}+e_{it}$$

Vector error $\varepsilon_{it}={\left(\mu_{it},e_{it}\right)}^{\prime }$ is stationary.

$\Omega_{i}$- asymptotic covariance matrix of vector error$x_{i}\;and\;y_{i}$ cointegrate for each cross-section with cointegrating vector β if $y_{it}$ presents only one unit root.

In our case, $\alpha_{i}$ allows the inclusion in the cointegrating equation of country fixed specific effects. Exogeneity of the regressors is not required and $x_{i}$ is a vector (m x 1) with uncorrelated values. $\varepsilon_{it}=\left(\mu_{it},{e_{it}}^{\prime }\right)$ is partitioned to have a constant as first element and a vector (m x 1) for the differences in the values of regressors $\varepsilon_{it}=\Delta x_{it}=x_{it}-x_{it-1}$.

Covariance matrix $\Omega_{i}$ can be written as:

$$\Omega_{i}=\left[\begin{array}{cc} \Omega_{11i} & \Omega_{21i}^{\prime }\\ \Omega_{21i} & \Omega_{22i} \end{array}\right]$$

$\Omega_{11i}$ is scalar long term variance corresponding to residual $\mu_{it}$.

$\Omega_{21i}$ is vector (m x 1) counting for the long-run covariance between $e_{it}$ and $\mu_{it}$.

$\Omega_{22i}$ is m x m long term covariance among the $e_{it}$.

The Asymptotic Bias of the Panel OLS Estimator of the coefficient β under invariance principle and cross-sectional independence is given by equation (3):

(3) $${\hat{\beta }}_{NT}={\left[\sum_{i=1}^{N}\sum_{t=1}^{T}{\left(x_{it}-{\bar{x}}_{i}\right)}^{2}\right]}^{-1}∙\sum_{i=1}^{N}\sum_{t=1}^{T}\left(x_{it}-{\bar{x}}_{i}\right)\left(y_{it}-{\bar{y}}_{i}\right)$$

${\bar{x}}_{i},{\bar{y}}_{i}$- individual means

The Asymptotic Distribution of the Pooled Panel FMOLS Estimator of the coefficient β is given in equation (4):

(4) $${\hat{\beta }*}_{NT}-\beta ={\left[\sum_{i=1}^{N}{\hat{L}}_{22i}^{-2}\sum_{t=1}^{T}{\left(x_{it}-{\bar{x}}_{i}\right)}^{2}\right]}^{-1}\sum_{i=1}^{N}{\hat{L}}_{11i}^{-1}{\hat{L}}_{22i}^{-1}\left(\sum_{t=1}^{T}\left(x_{it}-{\bar{x}}_{i}\right)\mu_{it}^{*}-T{\hat{\gamma }}_{i}\right)$$

$$\mu_{it}^{*}=\mu_{it}-\frac{\hat{L_{21i}}}{\hat{L_{22i}}}\Delta x_{it}$$

$${\hat{\gamma }}_{i}\equiv \hat{\Gamma_{21i}}+{\hat{\Omega }}_{21i}^{0}-\frac{\hat{L_{21i}}}{\hat{L_{22i}}}\left(\hat{\Gamma_{22i}}+{\hat{\Omega }}_{22i}^{0}\right)$$

${\hat{L}}_{i}$ Is lower triangular decomposition of ${\hat{\Omega }}_{i}$

Under invariance principle and cross-sectional independence we have:

$$T\sqrt{N}\left({\hat{\beta }}_{NT}^{*}-\beta \right)∼N\left(0,v\right)$$

v = 2, if ${\bar{x}}_{i}={\bar{y}}_{i}=0$ and v = 6 in rest of the cases, where N→∞,T→∞.

DOLS estimator presents the same asymptotic distribution such as FMOLS estimator. If cointegration is not detected, an autoregressive distributed lag dynamic panel model might be constructed.

Let us assume one model of this type of orders (p, $q_{1}$, …, $q_{k}$). For N cross-sections (i = 1, …,N) and T periods (t = 1,..,T), the form of the model is given in specification (5):

(5) $$y_{it}=\sum_{j=1}^{p}\lambda_{ij}y_{i,t-j}+\sum_{j=0}^{q}\delta_{ij}^{\prime }X_{i,t-j}+\mu_{i}+\varepsilon_{it}$$

$X_{it}$- vector of exogenous variables (k x 1)

$\lambda_{ij}$- scalars

$\delta_{ij}$- vector of parameters (k x 1)

$\mu_{i}$- group-specific effect

MG estimators are constructed in this study and they are unweighted means of individual parameters. This paper checks also for causality between variables using by Juodis, Karavias and Sarafidis test [26]. It starts from a linear dynamic panel data model denoted by (6):

(6) $$y_{i,t}=\varphi_{0,i}+\sum_{p=1}^{P}\varphi_{p,i}y_{i,t-p}+\sum_{p=1}^{P}\beta_{p,i}x_{i,t-p}+\varepsilon_{i,t}$$

i = 1,2, …,T; t = 1,2, …,N; p = 1,2,..,P.

$\varphi_{0,i}$- individual-specific effects

$\beta_{p,i}$- heterogeneous feedback parameters

$\varphi_{p,i}$- heterogeneous autoregressive coefficients

$\varepsilon_{i,t}$- errors

The null hypothesis states that $x_{i,t}$ does not Granger cause $y_{i,t}$.

$H_{0}:\beta_{p,i}=0$ , for all i and p

The alternative hypothesis supposes:

$H_{1}:\beta_{p,i}\neq 0$ , for some i and p

The analysis of correlation matrix in Appendix 1 revealed quite strong correlation between GDP per capita in the previous period and the following variables (Pearson’ coefficients of correlation in brackets): TNR (−0.4146), CR (−0.5470), NRG (0.4427), NGA (0.6354), CI x NGR (0.6744), CI x TNR (0.4437), CI x CA (0.6040), CI x NGA (0.7428). BM is strongly correlated with DC (0.7438), while DC is strongly correlated with MK (0.8542). Table 3 presents some descriptive statistics for variables in natural logarithm.

**Table 3:** Descriptive statistics (values in natural logarithm).

Variable	Mean	Std. Dev.	Minimum	Maximum
FD	−0.5522551	0.3554393	−1.291469	−0.1064345
FI	−0.6802127	0.484272	−1.610114	−0.0966554
FM	−0.4945168	0.3199862	−1.492211	−.0712534
GDP per capita	−1.759294	0.6899264	−2.980989	−0.8963366
CI	1.405391	0.5406434	0.4105992	2.025601
DC	4.354424	0.7245794	2.823625	5.331127
BM	4.366174	0.5268375	3.174203	5.335968
MK	28.56298	1.429174	26.48762	31.10052
TNR	1.578014	1.453961	−1.449454	4.016803
CR	−1.211862	0.9960877	−2.703673	1.599814
OR	0.8546388	1.915999	−4.788731	3.998128
NGR	−0.6034794	1.503578	−4.941601	2.160279
TNRA	11.25793	1.450228	8.110903	13.87519
CA	8.430359	1.159142	4.96192	10.53476
OA	10.53557	1.844937	6.159676	13.85651
NGA	20.14513	2.734594	13.52898	23.89117

The financial indexes registered a decrease in the analyzed panel. The maximum FD was registered in the USA in 2015, while the minimum value was reached by Arabia Saudi in 1996. Russia registered the lowest FI and FM in the panel in 1996, while the highest value of FI belongs to China in 2012 and the highest FM was registered by USA in 1999.

## 3 Results, discussion and robustness check

Preliminary tests like those for cross-sectional dependence, slope heterogeneity, unit root and cointegration are applied before the selection of the panel data model. According to Table 4 that shows the results of Pesaran's CD test, cross-sectional dependence is assumed for all variables at 1% significance level, excepting CI.

**Table 4:** The results of Pesaran's CD cross-sectional dependence test.

Variable	Stat.	p-value
FD	9.92*	0.000
FI	7.53*	0.000
FM	2.81*	0.005
GDP per capita	−3.01*	0.003
CI	0.72	0.469
DC	8.13*	0.000
BM	8.42*	0.000
MK	3.15*	0.002
TNR	11.01*	0.000
CR	9.08*	0.000
OR	10.34*	0.000
NGR	3.21*	0.001
TNRA	9.81*	0.000
CA	9.71*	0.000
OA	7.98*	0.000
NGA	3.47*	0.001

Note: * means significance at 1%.

Under the null hypothesis, the slope coefficients are homogenous in the Pesaran and Yamagata test [27]. This hypothesis is checked for all variables excepting TNRA and NGA as Table 5 shows.

**Table 5:** The results of slope heterogeneity test (Pesaran and Yamagata test).

Variable	Delta	Adjusted delta
FD	−0.899 (0.369)	−0.971 (0.332)
FI	−1.145 (0.252)	−1.237 (0.216)
FM	−0.607 (0.544)	−0.655 (0.512)
GDP per capita	−0.683 (0.495)	−0.738 (0.461)
CI	−0.453 (0.650)	−0.490 (0.624)
DC	−0.230 (0.818)	−0.252 (0.801)
BM	1.121 (0.262)	1.226 (0.220)
MK	−1.008 (0.314)	−1.127 (0.260)
TNR	−0.081 (0.935)	−0.088 (0.930)
CR	−0.730 (0.465)	−0.789 (0.430)
OR	−1.418 (0.156)	−1.532 (0.126)
NGR	−1.418 (0.156)	−1.532 (0.126)
TNRA	4.978* (0.000)	5.636* (0.000)
CA	−0.708 (0.479)	−0.764 (0.445)
OA	−1.202 (0.229)	−1.299 (0.194)
NGA	4.207* (0.000)	4.614* (0.000)

Note: p-values in brackets, * means significance at 1%.

The results in Table 6 based on CADF unit root test indicate that the data for the all of the variables are stationary in the first difference at 5% significance level. The equations corresponding to CADF test are augmented by one and two lags, since this test is sensitive to the number of lags. For data in level, trend is considered. Therefore, the cointegration relationship could be checked for data in level.

**Table 6:** The results of CADF test.

Variable	Data series in level (constant and trend)		Data series in the first difference (constant)	
	Augmented by one lag	Augmented by two lags	Augmented by one lag	Augmented by two lags
FD	−2.324** (0.010)	−3.108* (0.001)	–	–
FI	1.496 (0.933)	0.703 (0.759)	−3.998* (0.000)	−3.199* (0.001)
FM	−2.363* (0.009)	−1.517*** (0.065)	–	–
GDP per capita	−5.171* (<0.01)	9.975 (0.999)	−6.499 * (<0.01)	3.936* (<0.01)
CI	−2.571 * (0.005)	10.580 (0.999)	−4.607 * (<0.01)	−3.879 * (<0.01)
DC	−3.257* (0.001))	10.580 (0.999)	−4.074 * (<0.01)	3.614 * (<0.01)
BM	−5.105 * (<0.01)	10.580 (0.999)	−6.848 * (<0.01	−2.115 (0.001)
MK	−3.293 * (<0.01)	10.580 (0.999)	−4.851 * (<0.01)	3.414 * (<0.01)
TNR	−2.118** (0.017)	10.580 (0.999)	−3.652 * (<0.01)	3.614 * (<0.01)
CR	−0.747 (0.227)	10.580 (0.999)	−3.193 * (0.001)	3.614 * (<0.01)
OR	0.151 (0.560)	10.580 (0.999)	−1.790** (0.037)	3.614 * (<0.01)
NGR	−2.754 * (0.003)	10.580 (0.999)	−4.861 * (<0.01)	3.614 * (<0.01)
TNRA	−2.477* (0.007)	10.580 (0.999)	−3.199* (0.001)	3.614 * (<0.01)
CA	−1.233 (0.109)	1.200 (0.885)	−3.715 *(<0.01	3.024*(<0.01)
OA	−1.584*** (0.057)	−1.479*** (0.070)	–	–
NGA	0.638 0.738	2.606 0.995	−2.131* (0.001)	2.805*(<0.01)
CI X TNR	−0.729 0.233	−1.909 0.028	−4.082 0.000	3.190*(<0.01)
CI X CR	−1.397*** 0.081	1.390 0.918	−3.746*(<0.01)	4.634*(<0.01)
CI X OR	−0.408 (0.342)	−1.750 0.040	−3.131 0.001	3.845*(<0.01)
CI X OA	−1.278 (0.101)	−1.036 0.150	−3.248 0.001	3.992*(<0.01)

For non-stationary data in level, Westerlund test is applied to check for cointegration. According to results based on Westerlund test in Table 7, there are cointegration relationships from CA, GDP per capita, BM, MK to FI and from NGA, GDP per capita, BM, MK to FI. Therefore, DOLS and FMOLS model are estimated in this case.

**Table 7:** The results of Westerlund test for cointegration.

Statistics	Cointegration between FI and:			
	CA, GDP, DC	NGA, GDP, DC	CA, GDP, BM, MK	NGA, GDP, BM, MK
Gt	−0.3283 (0.3713)	−0.7475 (0.2274)	−1.4549 (0.0728)	−1.4549 (0.0728)
Ga	−1.8038 (0.0356)	−1.1311 (0.1290)	−1.5094 (0.0656)	−1.5094 (0.0656)
Pt	−0.7612 (0.2233)	−0.2736 (0.3922)	−0.8815 (0.1890)	−0.8815 (0.1890)
Pa	−1.5734 (0.0578)	−0.6241 (0.2663)	−1.5323 (0.0627)	−1.5323 (0.0627)

According to results in Table 8, the models based on CA indicate that CA has a negative impact on FI, while GDP per capita and BM exert a positive influence on FI. The models including NGA as explanatory variable suggest that GDP per capita and BM have a positive effect on FI and MK a negative one. NGA does not have a significant impact on FI.

**Table 8:** DOLS and FMOLS models to explain FI.

DOLS approach					FMOLS approach			
Variable in the previous year	Coefficient	p-value	Coefficient	p-value	Coefficient	p-value	Coefficient	p-value
CA	−0.0318961	0.000	–	–	−0.004359	0.0708	–	–
GDP_cap	0.5954367	0.000	0.0308902	0.0661	0.213972	0.0719	0.207774	0.0115
BM	0.5327608	0.000	0.1342591	0.000	0.144815	0.0138	0.106423	0.0177
MK	−0.0105702	0.374	−0.0131479	0.021	−0.008343	0.4338	−0.018755	0.0632
NGA	–	–	0.0003783	0.876	–	–	0.026031	0.1820

For non-stationary and non-cointegrated panel data series, MG (mean group) estimator is used to explain FD and FM. Only the valid models were selected and Table 9 presents the MG estimators used to explain FD. GDP per capita growth has not any significant impact on FD. TNRA, DC and CI x OA have a positive influence on FD at 10% significance level. BM is positively correlated with FD at 1% significance level, while OR, CI and MK in model M5 have a positive impact of FD at 5% significance level.

**Table 9:** MG estimators to explain FD in top 5 countries (1996–2021).

Variable in the previous year for:	Coefficients (p-values in brackets)						
	M1	M2	M3	M4	M5	M6	M7
GDP per capita	−0.3079335 (0.328)	−0.3672271 (0.184)	−0.3089487 (0.304)	−0.1764949 (0.518)	−0.2319977 (0.623)	−0.3138024 (0.229)	−0.3908095 (0.176)
TNRA	0.1065755*** (0.073)	0.0350191** (0.020)	–	–	–	–	–
DC	0.2092899** (0.029)		0.2090103** (0.042)	0.229252** (0.018)	–	0.216102*5 (0.001)	0.1672113** (0.032)
BM	–	0.185241* (0.000)	–	–	−0.0638259 (0.710)	–	–
MK	–	0.0208471 (0.322)	–	–	0.04377** (0.026)	–	–
OR	–	–	–	0.0932821** (0.024)	–	–	–
CI	–	–	–	–	0.3081454 (0.661)	0.118852** (0.037)	–
CI XOA	–	–	–	–	–	–	0.0974973*** (0.067)
Constant	−3.067183* (0.007)	−2.579944 ** (0.029)	1.114825* (0.008)	−1.813531* (0.003)	−2.053926*** (0.082)	−2.306243* (0.001)	−3.059918* (0.010)

Excepting M′6 when the influence is negative, GDP per capita growth does not significantly impact FM. The variables TNRA, DC in the model M′6, CA, OA, OR, CI x TNR and CI x OA in the previous period have a significant and positive effect on FM at 10% significant level. On the other hand, BM in the model M′2 positively impacts FM at 1% significance level. The results are presented in Table 10.

**Table 10:** MG estimators to explain FM in top 5 countries (1996–2021).

Variable in the previous year for:	Coefficients (p-values in brackets)						
	M’1	M’2	M’3	M’4	M’5	M’6	M’7
GDP per capita	−0.452549 (0.407)	−0.8555323 (0.111)	−0.4946927 (0.344)	−0.2706925 (0.545)	−0.862383** (0.030)	−0.4710635 (0.264)	−0.6077196 (0.225)
TNRA	0.2357182*** (0.093)	–	–	–	–	–	–
DC	0.2189221 (0.262)	–	0.2098354 (0.341)	0.2919815 (0.185)	–	0.252444***1 (0.055)	0.1224827 (0.480)
BM	–	0 .1939282* (0.000)	–	–	−0.2246097 (0.452)	–	–
MK	–	0.0102508 (0.881)	–	–	0.0624623 (0.218)	–	–
CA	–	0.0482602*** (0.081)	–	–	–	–	–
OA	–	–	0.212907***9 (0.065)		–	–	–
OR	–	–	–	0.240307** (0.041)	–	–	–
CI	–	–	–	–	−0.6354372 (0.618)	–	–
CI X TNR	–	–	–	–	–	0.276133*** (0.064)	–
CI XOA	–	–	–	–	–	–	0.19147***46 (0.060)
Constant	−4.69537** (0.022)	−3.331093*** (0.099)	−4.410958** (0.025)	−2.390426** (0.032)	−0.9183411 (0.567)	−3.157197* (0.002)	−4.232964** (0.013)

According to Table 11, Juodis, Karavias and Sarafidis (2021) Granger non-causality test indicates the following types of causalities between variables.

● Bidirectional causality between FD and NGA, CI, DC, BM, between FI and NGA, CI, CR_GDP, between FM and GDP per capita, CA, NGA, BM;
● Unidirectional causality from GDP per capita to FD, TNRA to FD, MK to FD, FD to CA, FI to GDP per capita, BM to FI, FI to CA, FI to DC, FI to MK, CI to FM, DC to FM, MK to FM, FM to TNRA, FM to CR_GDP.

**Table 11:** The results of Juodis, Karavias and Sarafidis (2021) Granger non-causality test.

Null hypothesis	HPJ Wald test	p-value
**GDP per Capita does not Granger-Cause FD**	10.301685*	0.0013
**TNRA does not Granger-Cause FD**	5.5425712**	0.0186
CA does not Granger-Cause FD	0.07358935	0.7862
OA does not Granger-Cause FD	2.6656702	0.1025
**NGA does not Granger-Cause FD**	4.8411627**	0.0278
**CI does not Granger-Cause FD**	26.864367*	0.0000
**CR_GDP does not Granger-Cause FD**	3.4095598***	0.0648
OR_GDP does not Granger-Cause FD	2.5053395	0.1135
**DC does not Granger-Cause FD**	28.94118*	0.0000
**BM does not Granger-Cause FD**	27.135116*	0.0000
**MK does not Granger-Cause FD**	21.662025*	0.0000
FD does not Granger-Cause GDP per Capita	0.10312937	0.7481
FD does not Granger-Cause TNRA	1.0435146	0.3070
**FD does not Granger-Cause CA**	8.3682965*	0.0038
FD does not Granger-Cause OA	0.49974371	0.4796
**FD does not Granger-Cause NGA**	7.7580381*	0.0053
**FD does not Granger-Cause CI**	13.080862*	0.0003
FD does not Granger-Cause CR_GDP	0.00026025	0.9871
FD does not Granger-Cause OR_GDP	0.00936047	0.9229
**FD does not Granger-Cause DC**	8.7061477*	0.0032
**FD does not Granger-Cause BM**	4.0379446**	0.0445
FD does not Granger-Cause MK	0.04025579	0.8410
**GDP per Capita does not Granger-Cause FI**	3.3530843***	0.0671
TNRA does not Granger-Cause FI	1.0144023	0.3139
CA does not Granger-Cause FI	0.21438894	0.6433
OA does not Granger-Cause FI	0.0198694	0.8879
**NGA does not Granger-Cause FI**	30.322453*	0.0000
**CI does not Granger-Cause FI**	39.9037*	0.0000
**CR_GDP does not Granger-Cause FI**	3.1436497***	0.0762
OR_GDP does not Granger-Cause FI	0.00274494	0.9582
**FI does not Granger-Cause GDP per Capita**	13.836324*	0.0002
DC does not Granger-Cause FI	0.02970534	0.8632
**BM does not Granger-Cause FI**	4.7721899**	0.0289
MK does not Granger-Cause FI	5.5358176**	0.0186
FI does not Granger-Cause TNRA	0.06247723	0.8026
**FI does not Granger-Cause CA**	12.100422*	0.0005
FI does not Granger-Cause OA	0.2729854	0.6013
**FI does not Granger-Cause NGA**	11.863663*	0.0006
**FI does not Granger-Cause CI**	28.571049*	0.0000
**FI does not Granger-Cause CR_GDP**	17.182811*	0.0000
FI does not Granger-Cause OR_GDP	1.5838254	0.2082
**FI does not Granger-Cause DC**	11.536491*	0.0007
FI does not Granger-Cause BM	1.8680454	0.1717
**FI does not Granger-Cause MK**	34.626914*	0.0000
**GDP per Capita does not Granger-Cause FM**	18.931591*	0.0000
TNRA does not Granger-Cause FM	0.64185977	0.4230
**CA does not Granger-Cause FM**	7.9768733*	0.0047
OA does not Granger-Cause FM	0.87460331	0.3497
**NGA does not Granger-Cause FM**	2.8474641***	0.0915
**CI does not Granger-Cause FM**	28.673973*	0.0000
CR_GDP does not Granger-Cause FM	0.19467245	0.6591
OR_GDP does not Granger-Cause FM	0.07560516	0.7833
**DC does not Granger-Cause FM**	18.291331*	0.0000
**BM does not Granger-Cause FM**	15.032117*	0.0001
**MK does not Granger-Cause FM**	17.805595*	0.0000
**FM does not Granger-Cause GDP per Capita**	3.0956053***	0.0785
**FM does not Granger-Cause TNRA**	8.4092973*	0.0037
**FM does not Granger-Cause CA**	17.688021*	0.0000
FM does not Granger-Cause OA	1.3163796	0.2512
**FM does not Granger-Cause NGA**	30.174972*	0.0000
FM does not Granger-Cause CI	0.00959502	0.9220
**FM does not Granger-Cause CR_GDP**	4.5279886**	0.0333
FM does not Granger-Cause OR_GDP	2.47572	0.1156
FM does not Granger-Cause DC	2.0319329	0.1540
**FM does not Granger-Cause BM**	12.86505*	0.0003
FM does not Granger-Cause MK	0.98065022	0.3220

The causality analysis indicated that there is a bidirectional causal relationship between each financial index and NGA. Economic growth supports the development of financial institutions in the top five gas exporters. The expansion of the economy makes companies to demand for more financial instruments and better access to external finance that are ensured by financial institutions. Companies in various fields offer more jobs, increase exports and require more sources of income. When an economy is expanding, banking system develops more. The role of growth in the development of financial institutions is supported also by previous studies [13,15].

More coal per capita contributes to the development of financial markets. Coal is considered the most carbon-intensive fossil fuel because of pollutant emissions and it has to be replaced by renewable energy sources. Even if policymakers and managers promote the “energy transition,” the global fossil fuel dependency tends to get worse. More than 80% of coal investment is made by six countries in the world (US, Canada, China, India, Japan, and the UK) [28]. The bidirectional causality between financial market development and coal per capita indicates that coal supports financial market, but also this market has mechanisms to entertain coal mining. This two-way causality explained the difficulties in achieving the transition from coal to renewable sources.

On the other hand, coal per capita had a negative influence on the development of financial institutions. The results are in line with [1,10] that explained how resource-rich countries tend to less developed from financial point of view, because the banks are more profitable and capitalized, but provide less credit to the private sector. Contract intensity measures “the importance of relationship-specific investment across industries” [29] and supports financial development. The interaction between contract intensity and oil per capita, respectively contract intensity and total natural resource per capita determines the development of financial markets.

Given these results, more policy recommendations could be formulated to ensure economic growth that supports the development of financial institutions in the top five gas exporters.

• Encouraging Competition: Implement policies that promote competition within the financial sector. This can be done by reducing barriers to entry, fostering innovation, and ensuring a level playing field for all financial institutions. By allowing more players in the market, economic growth can be better supported as different institutions bring in new ideas and services.
• Strengthening Regulatory Framework: Establish and enforce robust regulations and oversight mechanisms to ensure the stability and integrity of financial institutions. This can include measures to prevent fraud, improve transparency and accountability, and mitigate systemic risks. A strong regulatory framework provides confidence to investors and encourages the growth of financial institutions.
• Promoting Financial Inclusion: Implement policies that aim to increase access to financial services for all segments of the population. This can be achieved by improving financial literacy, expanding the reach of banking services, and facilitating the use of digital financial technologies. By enabling more individuals and businesses to participate in the formal financial system, economic growth can be more inclusive and sustainable.
• Supporting Infrastructure Development: Invest in the development of physical and digital infrastructure that supports the operations of financial institutions. This can include improving transportation networks, expanding broadband connectivity, and enhancing cybersecurity measures. By providing a robust infrastructure, financial institutions can operate more efficiently and effectively, contributing to overall economic growth.
• Encouraging Foreign Direct Investment: Implement policies that attract foreign direct investment (FDI) in the financial sector. This can be done by offering incentives to foreign investors, streamlining regulatory processes, and ensuring a favorable business environment. FDI can bring in capital, technology, and expertise, which can help strengthen and develop domestic financial institutions, leading to enhanced economic growth.
• Promoting Financial Sector Collaboration: Encourage collaboration between financial institutions, government agencies, and other stakeholders. This can include initiatives such as public-private partnerships, knowledge sharing platforms, and industry associations. By fostering collaboration, financial institutions can leverage each other's strengths, share best practices, and collectively contribute to economic growth.
• Investing in Human Capital: Support the development of a skilled workforce within the financial sector. This can be done through education and training programs, scholarships, and internships. By investing in human capital, financial institutions can have a competent and knowledgeable workforce that can drive innovation, adapt to changing market dynamics, and contribute to sustainable economic growth.

## 4 Conclusions

Despite the importance of the topic and the significant attention assigned to it by researchers, the contribution of natural resources to the financial development remains ambiguous and requires further research. Previous studies highlighted both negative and positive influence of natural resources on financial and economic development [30,31]. However, the results remain dependent on the proxies used to measure financial development.

All in all, the results of this study indicated a positive impact of GDP per capita only on financial institution index. As expected, broad money has a positive influence on all financial development indicators. Coal per capita negatively affects financial institution index, but positively impacts financial markets index. Contract intensity and its interaction with oil per capita contributes to increase in financial development index, while only the interaction between contract intensity and oil per capita, and total natural resource per capita respectively positively impact financial markets index. Market capitalization reduces financial institution index, but increases financial development index. Even if natural gas per capita does not have a direct influence on financial development indicators, bidirectional causality was observed between natural gas per capita and each financial markets indicator. There is also a reciprocal causality between contract intensity and financial development index, and financial institution index respectively.

Besides these results for the top five gas exporters, the study presents few limitations. For example, a panel data analysis was conducted since the period analyzed is short because of the available data. The study is limited only to five countries, but it could be extended by adding other gas exporters. The panel data models include only a limited number of control variables, omitting also economic variables that could be relevant.

Therefore, future recommendations should target the limitations indicated above. A separate analysis for each country in the sample is necessary to formulate suitable policies at national level. Moreover, the extension of the group of countries in a sample by considering other states is recommended to identify patterns for formulating relevant policy proposals at global level. The panel data models could include additional variables like business regulations or gross fixed capital formation.

## Funding statement

This work is supported by the Scientific Grant Agency of the Ministry of Education, Science, Research, and Sport of the Slovak Republic and the Slovak Academy Sciences as a part of the research project 10.13039/501100006109VEGA 1/0590/22.

## Author contribution statement

Mihaela Simionescu: Conceived and designed the experiments; Performed the experiments; Analyzed and interpreted the data; Contributed reagents, materials, analysis tools or data; Wrote the paper.

Beata Gavurova: Conceived and designed the experiments; Wrote the paper.

## Data availability statement

Data will be made available on request.

## Additional information

No additional information is available for this paper.

## Declaration of competing interest

The authors declare that they have no known competing financial interests or personal relationships that could have appeared to influence the work reported in this paper.

## Figures and Tables

**Table:** 

	year	country	fd	fi	fm	Ci	gfcf
year	1.0000						
country	0.0000	1.0000					
fd	0.2897	0.0056	1.0000				
fi	0.2329	0.1109	0.9298	1.0000			
fm	0.3082	−0.1312	0.8965	0.6743	1.0000		
ci	0.1293	−0.3178	0.2331	0.1450	0.3391	1.0000	
gfcf	0.2665	0.6407	0.5617	0.5785	0.4173	−0.3766	1.0000
to	0.0298	0.3606	−0.7535	−0.7704	−0.5948	−0.3072	−0.0952
dc	0.1252	0.0420	0.7846	0.7939	0.6559	0.5413	0.3947
bm	0.1517	0.5903	0.5129	0.5866	0.3434	0.0185	0.7521
mk	−0.0703	−0.4700	0.7980	0.7539	0.7618	0.4981	−0.0731
tnr_gdp	−0.0630	−0.0061	−0.6941	−0.7252	−0.5524	−0.6256	−0.1806
cr_gdp	0.2474	0.1992	0.3208	0.2688	0.3240	−0.4960	0.4817
or_gdp	−0.0551	−0.0141	−0.7077	−0.7314	−0.5739	−0.6197	−0.2109
ngr_gdp	0.0027	−0.2777	−0.6036	−0.6535	−0.4099	0.2237	−0.6327
gdp_cap	−0.0033	−0.5006	−0.0379	−0.1135	0.1018	0.9315	−0.6878
tnra	0.0862	−0.4428	−0.6916	−0.7558	−0.4712	−0.1126	−0.5141
ca	0.4408	−0.4528	0.3454	0.2502	0.4661	0.5063	−0.0641
oa	0.0592	−0.3576	−0.7344	−0.7846	−0.5332	−0.2399	−0.4819
nga	0.1337	−0.5147	−0.5331	−0.5934	−0.3260	0.4192	−0.7327
ci_tnr	−0.0182	−0.1514	−0.7404	−0.8183	−0.5197	−0.3093	−0.3908
ci_cr	0.3062	−0.1479	0.2849	0.1741	0.4014	−0.0010	0.1657
ci_or	−0.0218	−0.1215	−0.7514	−0.8082	−0.5597	−0.3951	−0.3713
ci_ngr	−0.0145	−0.3414	−0.4859	−0.5592	−0.2766	0.5072	−0.6701
ci_tnra	0.1372	−0.5495	−0.5836	−0.6791	−0.3309	0.2534	−0.6354
ci_ca	0.3650	−0.5813	0.2263	0.1206	0.3883	0.7343	−0.2786
ci_oa	0.1051	−0.4662	−0.6810	−0.7599	−0.4422	0.0546	−0.6077
ci_nga	0.1159	−0.5266	−0.4571	−0.5294	−0.2464	0.5584	−0.7310
lgdp_cap	−0.0049	−0.4979	−0.0397	−0.1018	0.0818	0.9305	−0.6870
	to	dc	bm	mk	tnr_gdp	cr_gdp	or_gdp
to	1.0000						
dc	−0.6607	1.0000					
bm	−0.1760	0.7438	1.0000				
mk	−0.8542	0.8542	0.1535	1.0000			
tnr_gdp	0.6988	−0.8956	−0.5323	−0.8122	1.0000		
cr_gdp	0.0248	−0.1504	0.1225	−0.0348	0.4390	1.0000	
or_gdp	0.6920	−0.8933	−0.5456	−0.8126	0.9788	0.3157	1.0000
ngr_gdp	0.4033	−0.5865	−0.6524	−0.5249	0.5357	−0.0700	0.4837
gdp_cap	−0.2034	0.2647	−0.2823	0.3661	−0.4193	−0.5316	−0.4028
tnra	0.5979	−0.6826	−0.6347	−0.5731	0.7539	0.0315	0.7452
ca	−0.2012	0.3073	−0.0412	0.5976	−0.1957	0.4502	−0.2891
oa	0.6398	−0.7664	−0.6516	−0.6382	0.8220	−0.0017	0.8541
nga	0.2544	−0.4658	−0.6839	−0.2933	0.4091	−0.1291	0.3687
ci_tnr	0.7126	−0.8404	−0.6347	−0.7885	0.9354	0.2548	0.9122
ci_cr	−0.0336	−0.0013	−0.0258	0.1858	0.2466	0.8688	0.1156
ci_or	0.7086	−0.8704	−0.6329	−0.7837	0.9391	0.1676	0.9658
ci_ngr	0.2679	−0.3553	−0.5662	−0.3431	0.3033	−0.1876	0.2511
ci_tnra	0.4668	−0.4742	−0.6103	−0.3489	0.5068	−0.1535	0.5004
ci_ca	−0.1990	0.3400	−0.1282	0.6658	−0.3072	0.1887	−0.3743
ci_oa	0.5623	−0.6324	−0.6671	−0.4897	0.6568	−0.1636	0.6915
ci_nga	0.1798	−0.3397	−0.6234	−0.1837	0.2775	−0.1874	0.2369
lgdp_cap	−0.2108	0.2512	−0.2897	0.3722	−0.4146	−0.5470	−0.3964
	ngr_gdp	gdp_cap	tnra	ca	oa	Nga	ci_tnr
ngr_gdp	1.0000						
gdp_cap	0.4370	1.0000					
tnra	0.5811	0.1141	1.0000				
ca	0.0624	0.3724	0.4592	1.0000			
oa	0.5470	0.0015	0.9666	0.2171	1.0000		
nga	0.9188	0.6314	0.6571	0.2915	0.5895	1.0000	
ci_tnr	0.7315	−0.0889	0.8678	0.0222	0.8933	0.6695	1.0000
ci_cr	0.0870	−0.0668	0.3346	0.8054	0.1884	0.1572	0.2762
ci_or	0.6334	−0.1638	0.8349	−0.1352	0.9204	0.5652	0.9656
ci_ngr	0.9534	0.6713	0.4990	0.1765	0.4259	0.9431	0.5872
ci_tnra	0.6313	0.4489	0.9327	0.5326	0.8540	0.7706	0.7318
ci_ca	0.1502	0.6141	0.5377	0.9572	0.2834	0.4191	0.0059
ci_oa	0.6178	0.2821	0.9603	0.3490	0.9562	0.7176	0.8249
ci_nga	0.8781	0.7382	0.5914	0.3338	0.5052	0.9872	0.5779
lgdp_cap	0.4427	0.9952	0.1096	0.3494	−0.0007	0.6354	−0.0939
	ci_cr	ci_or	ci_ngr	ci_tnra	ci_ca	ci_oa	ci_nga
ci_cr	1.0000						
ci_or	0.1272	1.0000					
ci_ngr	0.0773	0.4608	1.0000				
ci_tnra	0.2556	0.6688	0.6468	1.0000			
ci_ca	0.6343	−0.1466	0.3172	0.6745	1.0000		
ci_oa	0.1587	0.8271	0.5755	0.9547	0.4796	1.0000	
ci_nga	0.1436	0.4601	0.9500	0.7539	0.4852	0.6717	1.0000
lgdp_cap	−0.1052	−0.1640	0.6744	0.4437	0.6040	0.2759	0.7428
	lgdp_cap						
lgdp_cap	1.0000						
							
